# The interdependence of relational and material wealth inequality in Pemba, Zanzibar

**DOI:** 10.1098/rstb.2022.0288

**Published:** 2023-08-14

**Authors:** Daniel Redhead, Emmanuel Maliti, Jeffrey B. Andrews, Monique Borgerhoff Mulder

**Affiliations:** ^1^ Department of Human Behavior, Ecology and Culture, Max Planck Institute for Evolutionary Anthropology, Deutscher Platz 6, 04103 Leipzig, Germany; ^2^ Consultant, PO Box 3178, Dar es Salaam, Tanzania; ^3^ Department of Anthropology, University of California at Davis, Davis, CA 95616, USA

**Keywords:** inequality, material wealth, relational wealth, social relationships, social networks

## Abstract

The extent of inequality in material wealth across different types of societies is well established. Less clear, however, is how material wealth is associated with relational wealth, and the implications of such associations for material wealth inequality. Theory and evidence suggest that material wealth both guides, and is patterned by, relational wealth. While existing comparative studies typically assume complementarity between different types of wealth, such associations may differ for distinct kinds of relational wealth. Here, we first review the literature to identify how and why different forms of relational wealth may align. We then turn to an analysis of household-level social networks (food sharing, gender-specific friendship and gender-specific co-working networks) and material wealth data from a rural community in Pemba, Zanzibar. We find that (i) the materially wealthy have most relational ties, (ii) the associations between relational and material wealth—as well as relational wealth more generally—are patterned by gender differences, and (iii) different forms of relational wealth have similar structural properties and are closely aligned. More broadly, we show how examining the patterning of distinct types of relational wealth provides insights into how and why inequality in material wealth remains muted in a community undergoing rapid economic change.

This article is part of the theme issue ‘Evolutionary ecology of inequality’.

## 1. Introduction

Substantial diversity in economic and social inequality has been observed across human societies. In many settings, a small set of individuals have disproportionate access to, and control over, a group’s material resources, while in other settings material resources are more equally distributed between group members [1,2]. Existing research has provided a strong foundation for understanding the myriad micro- and macro-level factors that drive such differences in material wealth inequality in contemporary market-based industrial societies—highlighting, for example, the roles of the endowment of wealth between generations [3], the influence of local institutions [4], and differences in income and production [1,5,6]. Complementary to this, evidence from emerging economies has emphasized the importance of three interlinked factors—production systems [7], economic defensibility [8] and intergenerational wealth transmission [9]—that similarly pattern material wealth inequality in the global south.

Differences in material wealth are attributable to processes at the macro, meso and micro levels. Differences in material wealth may have individual foundations (i.e. micro-level, e.g. differences in skill or education), which produce gross structure through meso-level processes (i.e. interactions or social relationships); or features of a system at the macro-level (e.g. production system) guide or constrain meso-level processes to produce observable differences in material wealth at the micro level [10–12]. The contributions of meso-level processes to material wealth inequality have received comparatively limited attention, especially in rural parts of the global south. Theory suggests that networks of social relationships are crucial axes upon which material wealth differences may rest [13,14]. Social relationships provide platforms for mobilizing the resources of others, with individuals being more motivated to provide material, political or informational support to those that they have social ties with (e.g. friends or family; [15–17]). Such networks may be structured by material wealth. This means that choice in social partners may be driven by aspirations to create relationships with wealthy others to access their resources, and wealthy individuals are potentially motivated to maintain such asymmetrical relationships to maintain positive reputations, reduce social tensions, or access the resources of others [18]. Thus, the individuals or households with the most material wealth may have the greatest *relational wealth* (i.e. the largest networks of social relationships or most lucrative network positions) within their community.

Relational wealth is not a homogeneous quantity. Different types of relational wealth may have different associations with material wealth. For instance, individuals may preferentially form certain types of relationships with those similar to themselves in material wealth (i.e. wealth *homophily*), as such relationships would be considered mutually beneficial (e.g. with higher chances of reciprocity), and this assortment by material wealth could increase material wealth inequality [19–22]. Antithetically, other types of relationships could preferentially form between individuals without considerations of material wealth—or between individuals of different wealth—and could act to reduce differences in wealth [20,23,24]. Understanding how different strands of relational wealth are patterned by material wealth may shed light on whether and how changes in macro-level factors impact material wealth inequality.

Here, we aim to provide a comprehensive description of the associations between material and relational wealth in a rural economy in the global south. We first review the extant literature that details the complementarity between certain forms of relational and material wealth, and further outline the mechanisms that may drive linkages between such forms of wealth (in §2). In §3, we turn to the methods of our empirical study on the associations between three different forms of relational wealth with material wealth and two indicators of status in a rural community in Pemba, Zanzibar. We analyse our data using a Bayesian network analysis framework to assess: (i) the associations that material wealth has with food sharing, friendship and co-working relations, (ii) how these different types of relational wealth are patterned across the community, and (iii) whether different types of relational wealth are complementary and overlap. We outline our results in §4. In the Discussion (§5), we interpret the significance of our results for the specific cultural context, as well as explore more generally the implications of our findings for understanding the interdependence between relational and material wealth in human communities.

## 2. Material and relational wealth

Material wealth inequality—defined as differential access to resources between individuals or groups—has persisted over human history [25–27], and pervaded multiple cultural and ecological settings [7,28], often with major impacts on life outcomes. Most consistently, individuals with privileged access to material resources are often healthier [29–31], and have greater reproductive success (especially men; e.g. [32–36]). The ubiquity of material wealth inequality, and the importance of being wealthy, beg questions as to how inequality has emerged to become a fundamental characteristic of human social organization.

Focus has primarily been on the macro-level ecological processes and structural features that cause material wealth inequality to emerge. Anthropologists, in particular, focus on the environmental conditions that allow individuals differential access to material resources [37]. Specifically, climatic stability [38], circumscription [39], resource density and patchiness [40], storage [41] and institutions for inter-generational transmission [9] can produce pay-off structures for individuals that encourage the privatization of resources [42–44], thereby giving rise to inequality [8]. When such dynamics are coupled with an analysis of micro-level factors, we can learn about who becomes rich and why. Individuals with embodied wealth (e.g. skill, knowledge, strength, health; [45]) may increase their production or control of resources. These effects may be direct, through simply grabbing wealth by threatening harm, but often operate through informal social influence, whereby an individual’s perceived ability and willingness to provide benefits improves their status within a community, at times strengthening hierarchical structures [46–48]. Akin to this, leadership roles often accompany expectations of making decisions in the best interest of a group [49], and in turn greater access to the material goods that are available to their group [50]. As such, social hierarchy and leadership can exacerbate material wealth inequality within a group.

This macro and micro-level framing of inequality leaves unaddressed the precise processes whereby inequality emerges and is maintained. Meso-level analysis of interactions and social relationships, typically through network analysis [18,51], provides a useful tool in this regard. Following many who recognize the critical nexus between relational and material wealth (e.g. [20]), we review and empirically examine how the study of social networks can shed light on the differing associations between relational and material wealth, which might exacerbate or mitigate material inequality. More specifically, we examine the situations where the wealthy choose to associate primarily with one-another, where the wealthy may find it in their interests to establish patron–client relationships with less wealthy others, and where challenges contingent on collective action and/or resource unpredictability mute the ability of the wealthy to withdraw from the whole community. More fundamentally, we explore the validity of a core assumption underlying studies of the persistence of inequality that material and relational wealth are necessarily complementary [9,52].

Observable differences in material wealth may have positive, negative, or no clear association with relational wealth, and the materially wealthy may or may not chose to preferentially associate. In communities where there is increasing focus on defending private resources, and consequently less reliance on social support, wealthier individuals may be particularly selective about engaging in certain types of relationships [22]. They may do this not only to protect their material wealth, but also to benefit from ties with others in possession of similarly important material, informational or social resources [20]. Such friendships will be mutually beneficial for both parties, entailing exchanges of similarly valued resources, and often with the relaxation of obligations that characterize cooperation (e.g. no expectations of direct reciprocity or strict book-keeping; [17,53]). The rich will find such ties advantageous (especially when disadvantaged groups are large; [54]). These preferences can amount to social networks becoming structured by wealth homophily [21,55]. Furthermore, homophily can become self-reinforcing insofar as wealthy individuals—with greater access to valued material, social and informational resources—become increasingly valuable as partners [24,56]. The resulting wealth gap can be bolstered by preferences for, or assortment by, personal characteristics that signpost shared norms (e.g. religion; [57]), or by perceived badges of status (e.g. discrimination based on gender or ethnicity that can further entrench poverty traps; [18,58–60]). Such preferential assortment by wealth can inhibit upward mobility, prevent learning, and create clustering and separation in networks based on relative wealth [61]. Individuals who are less wealthy can only mobilize the resources that their similarly impoverished friends have access to, which are likely less valuable and diverse in comparison to wealthier counterparts [62,63]. Given this, homophily based on preferential assortment by wealth may be a leading factor in creating and sustaining wealth inequality, and will leave a signature on the networks of some communities.

In some contexts, wealthier individuals may be motivated to form asymmetrical patron–client relationships [64–68], or be well-positioned to broker relationships between other group members [69–72]. This is because they are either in a position to extract resources from others, and/or have disproportionate control over resources that other group members lack. In such situations, the non-wealthy may try to gain access to resources through social relationships, wage labour, or co-working ties, and we might expect to see ties typical of patron–client relationships, such as labour being sent from the poor to the wealthy and food (or other benefits) from the wealthy to the poor. The realization of such relationships nevertheless rests upon the decisions of wealthier group members, who may prefer to retain their isolation given their economic independence [73]. The extent to which wealthier individuals might be allowed to occupy network positions that allow them to maximize personal gains, and go unchecked by other group members, could depend on the strength of relationships among the non-wealthy and their ability to mount challenges to the status quo [74,75].

There are, however, constraints on the wealthy protecting their privilege. This is particularly the case when there is a high premium on coordinating group activities, such as managing subsistence tasks (e.g. foraging, livestock management; [65,76]), reducing costly intra-group conflict [23], succeeding in inter-group conflicts [77], or managing risk [42]—linking back again to macro-level features in the environmental and institutional context. Such problems require effective collective action and coordination [78] and would preclude a rich elite from entirely withdrawing their ties from the poor. Under such circumstances, we would again expect to see little patterning of social networks by wealth homophily.

The specific meso-level mechanisms that generate inequality under given macro and micro level conditions are difficult to recover from studies covering large time scales, or those addressing issues of broad geographical scope. Studies of contemporary populations nevertheless provide an excellent opportunity to unpick these meso-level processes, specifically through day-to-day inter-individual and inter-household interactions. Accordingly, we designed the analyses presented below to describe how material and relational wealth are related. We use data from a community that relies on cloves, a cash crop with yields that are highly variable both between households and overtime within households, and where little cash circulates in the village. In such a context, we might anticipate an important role for relational wealth buffering the vagaries of material affluence. More specifically, material wealth is created primarily by planting and harvesting clove (and other) agro-forestry products (often spices). The large but highly unpredictable cash windfalls appear, at least on ethnographic observation, to be invested in, and buffered by, relational wealth. This renders households to be highly-interdependent, with ties extending far beyond kin networks. At the time of study, there was little evident material inequality in lifestyles, despite considerable inequality in clove tree holdings (overall Gini in cash value of household wealth 0.38, Gini for clove tree value 0.62) and status differences were limited. With a recent dramatic rise in the price of cloves on the global market, we anticipate growing inequality. Using cross-sectional data to describe whether and how this material inequality is patterned by relational wealth, and how such ties differ for men and women, we hope to identify potential pathways whereby inequality can become either entrenched through wealth homophily, or mitigated through ties with households of differing wealth. Furthermore, we examine these dynamics using different forms of relational wealth—specifically in the domains of food sharing, friendship and co-working—because of their potential to be very differently patterned by material wealth considerations.

## 3. Methods

### (a) Ethnographic setting

Pemba is the northern of the two main islands of the Zanzibari archipelago. It lies 50 km off the coast of Tanzania at approximately 5°10’ S, 39°47’ E and spans 1014 km^2^. The island’s deep historical links with the Middle East have dramatically shaped its economy [79]. With a population density of $428\;{\text{individuals km}}^{2}$ and a population growth rate of 3.1%, it is rapidly transitioning from a forest- and fisheries-dependent economy [80] to one of mixed urbanization. The study site, which we will refer to as Mitini for anonymity, originally settled by a small group of related men seeking a new life during the Pemba famine of 1971–1972, was selected for its heavy reliance on cloves and its isolation. The 44 households in Mitini (*n* = 300) lie within 1 km of the coast, and 2–3 km from the ward town through which a small feeder road runs, and where primary school and health facilities are available. The track to the ward town is often impassible, with parents keeping school-aged children at home because of the dangerous mud. Mitini has a basic shop, four mosques, a non-working water pump and no other services.

Cloves were initially introduced to Zanzibar by the Omani Sultanate in 1822. Unlike the clove plantations on Unguja (the southern Zanzibari island), production in Pemba remained largely in the hands of small-scale local producers [81], and in Mitini most clove labour is supplied by family, co-workers, and some paid labour (from co-villagers and outsiders). While the economic importance of cloves has varied dramatically over the last 200 years [82,83], prices paid by the government marketing board have been climbing since 2010. Locals now joke that cloves *can even make dogs rich*. Production has high inter-year temporal variation, is highly seasonal and is generally unpredictable. Notably, shocks are not only aggregate but idiosyncratic, resulting from the fragility of clove buds, and the vulnerability of clove trees to damage and disease, often contingent on harvesting accidents.

Staple crops in Pemba are cassava and rice, but production in Mitini is limited by soil types and slope. Rather, households appear to support themselves on profits from cloves and other tree and spice crops (mangos, cinnamon, pepper, orange, jackfruits, timber etc.), and a wide array of crafts, local medicines and services (religious, healing, henna-painting, carpentry, house and boat building). The village population is entirely Muslim, extremely culturally homogeneous (unlike many Pemban villages, there are no households with remittance-sending relatives in Oman or elsewhere). Residents are settled across four small hamlets of patrilocal households. Each such household constitutes the home of a woman, her children, and (in all but one case) her husband. Extended families are not common, insofar as married offspring typically set up their own households at marriage. Polygynously married men (six men had two wives at time of survey) are associated with multiple households (for treatment of their wealth, see below).

To examine the associations between material and relational wealth, we use *household-level* network data and material wealth inventories from all households in Mitini. These data were collected as part of the ongoing *ENDOW* project (https://endowproject.github.io). While our introductory discussion of the dynamics of material and relational wealth was at the individual level, analyses at the household level here are justified as: (i) an individual’s material wealth is largely a function of the material wealth of their household and, (ii) food sharing occurs between households—in line with many existing analyses of food sharing (e.g. [56,84–87]). Parsing individual-level heterogeneity within households goes beyond the scope of the current analysis.

### (b) Data

#### (i) Data collection

Data on male and female household heads were collected separately by E.M. and M.B.M., and two trained research assistants. Verbal consent was granted by each interviewee, and each was paid 5000/−(USD 2.1) in the form of mobile phone vouchers. Breaks were taken for prayers. The protocols ran smoothly with consistently high cooperation. Our primary methodological concern was that women typically nominated many more ties than did men. Because we gender-matched interviewers to interviewees, we are unable to adjust for the possibility that interviewers may contribute to this gender difference.

#### (ii) Relational wealth

**Figure 1.**
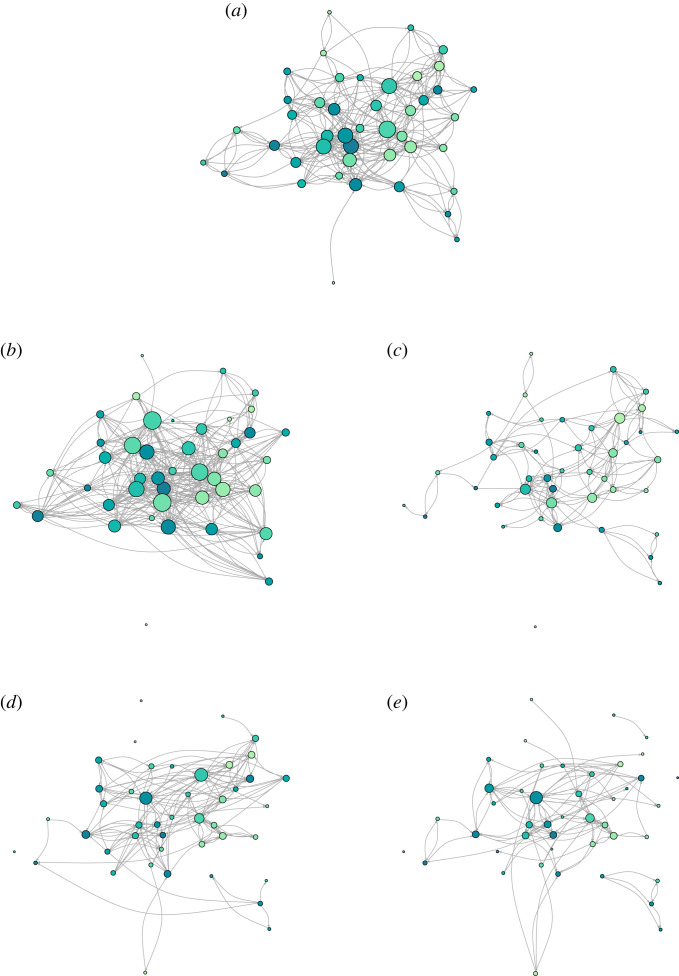
Network digraphs. Households (circles/nodes) are coloured by material wealth, with the darker and more blue colour showing households ranking higher in material wealth, while the lighter greener colours are assigned to those ranking lower in material wealth. Households are sized by the number of relationships that they have (both outgoing and incoming nominations). Household position within all digraphs was fixed by their position in the food sharing network. All digraphs were constructed using the igraph R package [90]. (*a*) Food sharing, (*b*) women’s friendships, (*c*) women’s co-working, (*d*) men’s friendships, (*e*) men’s co-working. (Online version in colour.)

To measure relational wealth, we construct social networks based on food sharing, friendship and co-working—with the latter two questions framed differently for men and women. As might be anticipated in a community founded on daily face-to-face interactions, ties of food-sharing, friendship and work capture the key social dynamics whereby households survive, persist and prosper, effectively a network of trusted partners who may not necessarily exchange cash in times of trouble. All questions were piloted in a neighbouring village. We measured food sharing through a double-sampled name-generator design. In doing this, we took two measurements of food sharing from the same participant, for each direction (i.e. ‘giver’ or ‘receiver’) of the relationship (see the electronic supplementary material for a more detailed explanation of our definition; see also [88,89]). This was done by first asking participants about which households share food, specifically: *which households commonly help you with food? For example, people who will bring food (prepared or unprepared) to your house?* We followed this with a question about the households that they share food with: *to which households do you often give food? For example, people to whom you will take food (prepared or unprepared) to their house?* All other measurements of relational wealth were single-sampled, directed networks. See figure 1 for visualizations of these networks, and the electronic supplementary material for further information and descriptive statistics.

#### (iii) Material wealth

We collected fine-grained inventory data for each household in our sample, effectively enumerating all material items that had monetary value. As noted above, we identified households as the primary sharing unit in the village, recognizing that there are intra-household inequities in resource access, and higher level entities conveying resource access—such as *wakf*, land held as a joint trust for family members under Islamic law. We treated polygynous households as decentralized [91], with each wife having her own household where she holds her own assets, plus a share of items that are culturally viewed as male property (e.g. trees, fishing tackle, carpentry equipment). Household inventories were given a point estimate of wealth by linking each possession to a cash value, based on the cost of replacement from a shop or other local source. For items with no clear cash value, primarily trees for which there is no cash market, we asked multiple respondents how much they might expect to receive for a specific tree species were they in desperate need of cash.

#### (iv) Status

There are no major social divisions within Mitini, no government officials or professionals, and divisions along ethnic or political lines—so salient in Zanzibar—were also not obvious. There are, however, some subtle differences in status, power and skills that might shape relationships and their influence on wealth, of which we captured two. First, we recorded whether any household member had spiritual or secular influence (typically holding a position of religious significance within the mosque or acting as an informal assistant to the ward government representative). Second, we noted whether any household member belonged to the village labour cooperative called *Nguvu Zetu* (*our strength*), which provides voluntary labour and cash to projects—such as communal tree planting or road maintenance—that is indicative of orientation towards development initiatives offered by the state or, more minimally, divergence from pure traditionalism.

#### (v) Genetic relatedness

Genealogical data were collected during kinship surveys through ascertaining the names of parents and both maternal and paternal grandparents. These genealogical data were used to calculate Wright’s coefficient of relatedness (*R*; [92]) for every pair of individuals in the sample (using the Kinship2 package in R; [93]). We then aggregated this individual-level data to the level of the household by taking the average *R* between households.

#### (vi) Physical distance

Coordinates for each physical household unit were recorded using a Columbus V 990 Mark II GPS unit. These coordinates were then used to calculate a normalized physical distance between households (using the Geosphere R package; [94]). See the electronic supplementary material for justification of including physical distance in the present analyses.

### (c) Analytical strategy

We modelled household-level (material wealth), dyad-level (genetic relatedness, physical distance, food sharing, women’s and men’s friendships and co-working relationships), and block-level (religious status, development group membership) covariates that may be related to the probability of network ties using a generalization of the social relations model [95], which integrates block-level random effects (i.e. a stochastic block model; see [96], for a technical outline and tutorial). We ran separate models for all single-sampled networks, and a latent network model [96,97] for our double-sampled food sharing network. We included all relational wealth types in our models to examine the overlap between these forms of relational wealth. We specified the same combination of parameters for each model to ensure comparability. Given the complexity of our models, we ran analyses with different specifications to assess how sensitive our main conclusions are to any different combinations of parameters. Results indicate that our main conclusions are robust to different model specifications. We cannot ascertain any causal inference with understanding the causal dynamics that link different network layers to one another and to the other demographic variables. However, our analysis describes associations that support the development of future generative causal models.

Given this, our model can generally be written as

$$A_{\left[i,j\right]}∼\mathrm{Bernoulli}(\mathrm{logistic}(\phi_{\left[i,j\right]})),$$

where

$$\begin{array}{rl} \phi_{\left[i,j\right]} & =\alpha +\lambda_{\left[i\right]}+\pi_{\left[j\right]}+\delta_{\left[i,j\right]}\\ & \;+\beta_{\left[1,S(i),S(j)\right]}+\beta_{\left[2,M(i),M(j)\right]}\\ & \;+\gamma_{1}W_{\left[i\right]}+\gamma_{2}{\bar{W}}_{\left[i,j\right]}\\ & \;+\gamma_{3}Q_{\left[i,j\right]}+\gamma_{4}K_{\left[i,j\right]}\\ & \;+\gamma_{5}R_{\left[i,j\right]}+\gamma_{6}D_{[i,j].} \end{array}$$

Here, we model the probability of a tie between two individuals in a given relational wealth network, *A*_[*i*,*j*]_, where *α* is an intercept term, *λ*_[*i*]_ is a vector of sender random effects, *π*_[*j*]_ receiver random effects, and *δ*_[*i*,*j*]_ are dyadic random effects. *β* are a list of block random effects for religious status, *S*, and membership to development group, *M*. *γ* is a vector of standard slope coefficients. We assume *A*_[*i*,*j*]_ to be a function of log wealth, *W*, dyadic log distance in wealth, $\bar{W}$, a combination of the other two relational wealth types, *Q* and *K* (e.g. if *A*_[*i*,*j*]_ was food sharing then *Q*_[*i*,*j*]_ would be co-working, and *K*_[*i*,*j*]_ friendship), spatial distance, *D*_[*i*,*j*]_, and relatedness, *R*_[*i*,*j*]_. For our double-sampled network, food sharing, we incorporated a measurement model that estimated and adjusted for biases—falsely reporting ties that are not there, forgetting true ties, and question duplication—typical to double-sampled network data (see [96] for further details). All analyses were implemented in R [98] using rstan, and the STRAND package for Bayesian social network analysis [96,99].

By applying this modelling framework, we are able to parse the effects of household characteristics (e.g. material wealth) that may be associated with each type of relational wealth, with effects of the attributes of a dyad (i.e. two households)—such as whether women in the households are friends or the physical distance between households. Alongside this, we assess whether households were likely to reciprocate ties (i.e. *dyadic reciprocity*), and whether households that made a larger number of nominations also received greater nominations (i.e. correlation between sending and receiving ties, referred to as *generalized reciprocity*).

Given the symmetry between sender, receiver and dyadic similarity terms for covariates in the social relations model, we include only two of the three terms in our analyses to ensure that the models are identifiable. As we are most interested in how material wealth is associated with propensities for *sending social support* and *how wealth homophily patterns social support relations*, we included sender and dyadic similarity terms to best reflect these forms of relational wealth.

Here, we considered ‘block-level’ covariates to be observed attributes that households can be meaningfully grouped into (e.g. religious status; [22]). In doing so, we answer questions about whether relational wealth flows to or from households with or households without members of these groups—groups that in Mitini are based on potential religious/administrative influence and a pro-development orientation.

## 4. Results

### (a) The associations between material and relational wealth

**Figure 2.**
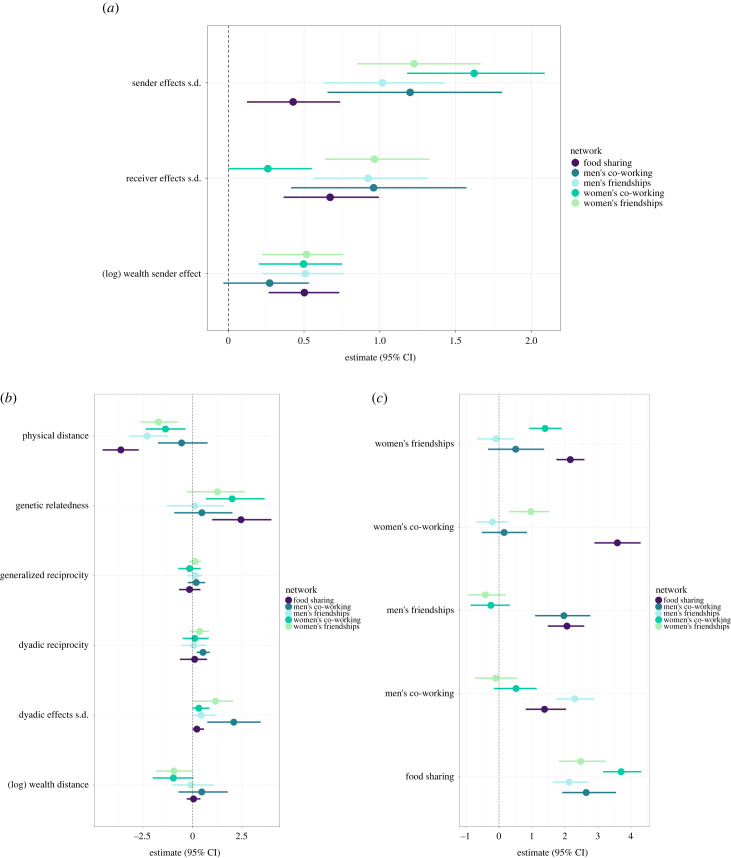
Standardized effects (posterior median and 95% credible intervals (CI)) of (*a*) household-level effects/covariates, (*b*) dyad-level effects/covariates, and (*c*) cross-network effect. Each bar represents a coefficient, and is coloured by relational wealth type. Positive estimates indicate (*a*) ego/household characteristics, or (*b*) dyad/shared household characteristics that are associated with an increased likelihood of sending that relational wealth type (e.g. sending food to another household). s.d., standard deviation. (Online version in colour.)

We find reliable associations between material and relational wealth. For women, those living in wealthier households reported having more friendships, and more co-working ties (figure 2*a*, (*log*) *wealth sender effect*) than those in poorer households. Further, these relationships were slightly more likely to be observed between households of similar wealth (as shown by the negative estimate for wealth distance on women’s friendships and co-working in figure 2*b*, (*log*) *wealth distance*). As shown in figure 2*a*, wealthier men were also more likely to report having more friends, and were marginally more likely to report having a higher number of co-working partners. Men’s friendships and co-working relationships were not reliably patterned by wealth homophily (i.e. wealth distance had no reliable association with the probability of forming ties; see figure 2*b*).

With respect to food sharing we find a similar pattern. We find a positive association between material wealth and food sharing. Wealthier households were, on average, more likely to share food with others (figure 2*a*, (*log*) *wealth sender effect*). There was no reliable association between wealth similarity and food sharing (figure 2*b*, (*log*) *wealth distance*).

### (b) The structure of relational wealth

As shown in figure 2*b* (*genetic relatedness*), food sharing, women’s friendships and women’s co-working relationships were patterned by genetic relatedness. This suggests that households were more likely to share food with kin, and women were more likely to be friends and work with their kin. By contrast, men work with, and form friendships with others, regardless of whether they are kin—as there was no reliable association with relatedness. Physical distance also patterned relational wealth (figure 2*b*, *physical distance*)—with food sharing, women’s co-working, and men’s and women’s friendships being more likely observed between households closer in physical space. Men’s co-working ties were not reliably associated with physical distance, suggesting that men tend to work with other men regardless of how close they live to one another.

**Figure 3.**
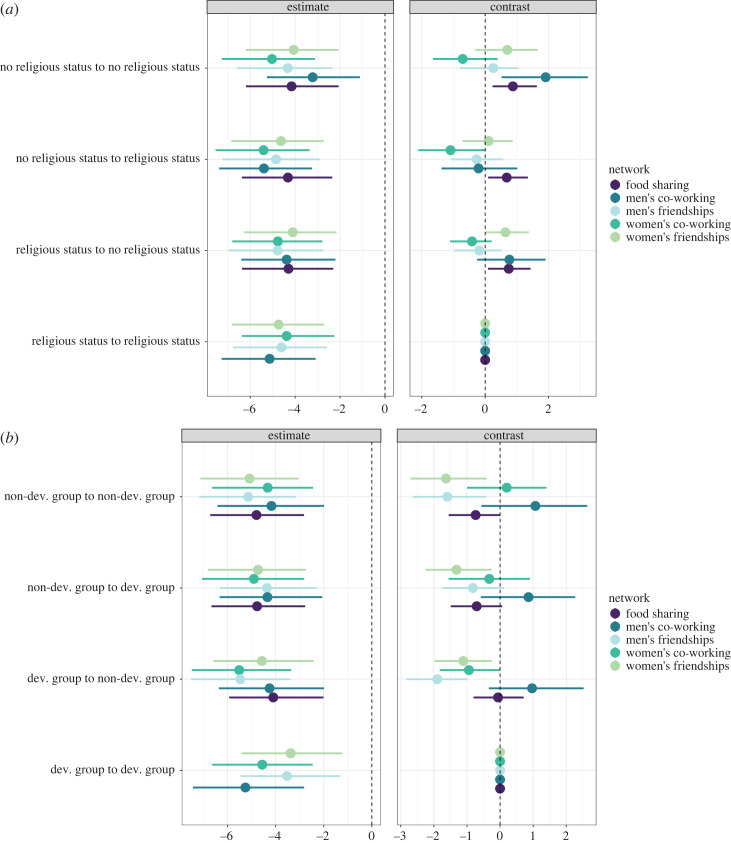
Offsets (95% credible intervals (CI)) and contrast coefficients, Δ (95% CI), for the interaction between (*a*) religious status and (*b*) development group membership on relational wealth of ego-alter dyads (i.e. pairs of individuals). Bars are coloured by relational wealth type. Δ here represents the change in probability of relational wealth ties between and within different levels of (*a*) religious status and (*b*) development group status, relative to the probability of ties between two individuals who have religious group or development status. Negative values—that do not encompass 0—indicate that relational wealth ties between individuals who both have status are reliably less likely than the parameter of interest, whereas positive coefficients indicate that they are more likely. (Online version in colour.)

To assess whether homophily based on status patterned relational wealth, we compute contrast coefficients, which assess whether ties between households with either a person of influence or development group membership are reliably more likely to be observed than ties between other membership combinations. We find that religious status plays little reliable role in patterning relational wealth, other than that men from households with no such status are more likely to work with each other. We find that food sharing is reliably less likely to be observed between individuals with religious status (figure 3*a*). Development group membership plays a more important role in patterning relational wealth. As shown in figure 3*b*, men and women who are members of the development group are reliably more likely to be observed as friends. Non-development group members are less likely than development group members to share food with one another, or with development group members.

We find heterogeneity in sending and receiving ties across all relational wealth measures (see the electronic supplementary material for details). The absence of reliable correlations in sending and receiving ties (see figure 2*b*, *generalized reciprocity*), indicates that (for example) households which share more food with others are no more likely to receive food. Similarly, there was an absence of a trend for households to reciprocate nominations across most relational wealth types (figure 2*b*, *dyadic reciprocity*). We discussed these findings further in the electronic supplementary material. Note the only exception to these trends was men’s co-working relationships, where some reciprocation was observed (figure 2*b*, *dyadic reciprocity*).

### (c) The associations between relational wealth types

We find that men’s and women’s relational wealth operate within different domains (figure 2*b*). Women’s friendship and co-working ties showed no reliable overlap with men’s friendship and co-working ties. That is, women were no more or less likely to report being friends or working with people in the same households as those that men in their households were friends or worked with. Alongside this, food was more likely to be shared between households where members (women or men) were either friends or worked together. This association was especially pronounced for women’s co-working relationships.

## 5. Discussion

Our network-based approach advances an empirical framework for understanding the meso-level processes that structure relational wealth. By going beyond simple correlations between different centrality metrics calculated on a network, we depart from existing approaches to the emergence of material wealth inequality. Instead, we consider the structure of different forms of relational wealth, and how such structure is connected to material wealth. Through this, we can explore empirically how material wealth and different types of relational wealth might interact to possibly exacerbate or reduce inequality. Focusing on a population with little evident material wealth inequality, we find that (i) the materially wealthy have more relational wealth ties, but not necessarily more ties with other wealthy people, (ii) different forms of relational wealth have similar structural properties and predominantly map onto food sharing, and (iii) despite these shared structural features, relational wealth is differently patterned for men and women, as well as by orientation to economic and social development.

### (a) The materially wealthy have more relational wealth ties

The material wealth of households has important associations with each type of relational wealth. Wealthy households help other households by sharing food more than do poorer households. This is not only because they have more to share, but because of the Islamic customary obligation of sharing with those of lesser fortune, particularly on Fridays and religious holidays. Indeed, the verse *Those who spend in charity will be richly rewarded* (Qur’an 57:10) is understood to mean that wealth is not diminished by charity, but rather enhanced through the spiritual rewards afforded to the donor—rewards also likely to affect the donor’s status. Men and women in wealthier households nominate more friends than those in poorer households, perhaps because they have more time to socialize—notably members of some of the poorest households in Mitini spend time working in roadside villages, thereby reducing their opportunities for socializing in the village. That women in wealthier households have more co-workers is probably attributable to their greater productive enterprises, with cassava, spices or tree planting. These positive associations are consistent with the assumption of complementarity between material and relational wealth.

Critically, however, we find no evidence of material wealth homophily in any measure of relational wealth for men (including food sharing)—an observation that suggests there is as yet little sub-division of the community into classes based on success in clove production, despite the huge windfalls that some families reap. This may be because wealthier households own hundreds of trees. Clove production is labour-intensive and an ideal source of wage-labour for poorer men and women alike. Wage-labour markets in informal economies such as in Mitini are often highly structured by other social networks (friendship and family), thereby both reinforcing friendships and working relations across all levels of society, and impeding the emergence of wealth homophily. Similarly we find no strong evidence that religious status structures social ties, although members of *Nguvu Zetu* are more likely to be friends. This may be indicative of possible emerging fissions around development orientation.

### (b) Different forms of relational wealth are similarly structured

While a number of advances have been made in understanding the structure of material wealth and its associations with embodied wealth [9], relational wealth—despite its intricate links with material inequality (as shown here; and see [20])—remains relatively ill-defined and ambiguous in the literature (as with notions of *social capital*; [100]). Furthermore, previous work on how relational wealth is linked to material wealth typically takes various network centrality statistics as indicative of ‘advantage’. Such approaches do not consider the reasons for how an individual or household has a specific position (i.e. as indicated by their centrality) within a networks, nor why such a position would be considered advantageous in the local context. As a result, the relevance of such centrality measures for relational wealth remains unclear. Our approach is different. We attempt to dissect the constituent parts of relational wealth, and to understand how these parts are linked together in the current ecological and cultural context.

We find food sharing to be fundamental to relational wealth in this community, insofar as it alone is consistently related to friendship and co-working networks—although the causal direction cannot be inferred in the present study. It is indeed normative to share food in Pemba, and food sharing is integral to work and friendship. For example, people often take food to family and friends working in agro-forestry plots. As in many cultures, food sharing, with all its symbolic significance, probably cements relationships across different domains and provides an important foundation for creating, sustaining and reflecting relational wealth [23,24,56,86].

In addition to food sharing, many of the different domains of relational wealth measured in this study showed strong overlap. Specifically, our data suggest that men and women were friends with those they worked with. These findings reflect daily life in a community that has little economic specialization and where individuals draw few social distinctions between the people with whom they work and play. Indeed, in Mitini, work and social activities often occur simultaneously—as, for example, when people chat or pray during work breaks. Furthermore, the pool of potential partners is small and somewhat spatially constrained across four distinct hamlets, such that there are not many social partners among whom to chose.

### (c) Gendered patterns of relational wealth

Genetic relatedness is a salient feature of womens’ social networks, with women reporting friendships and co-working relationships with closely-related individuals. Food is also shared preferentially between related households. For men, we see less emphasis on genetic relatedness. Kinship featured little in accounting for their work or friendship ties, and was replaced by reciprocity—at least for co-working ties. Note that we only analyse ties *within* Mitini. While it is possible that men’s co-working/friendship ties beyond the village are indeed patterned by kinship, we have no reason to expect that this would differ for women. Women and men are more similar with respect to the extent to which physical distance constrains their friendships and food-sharing. Notably, however, there is no relationship between physical distance and men’s co-working relationships. Furthermore, although women report many more relational ties than do men, this may reflect interviewer bias. Recall our interviewers were gender-matched, such that we cannot statistically adjust for any interviewer effects that could create differences between the number of men’s and women’s reported ties.

The way that gender differentially patterns relational wealth appears—at least superficially—to defy expectations for a strongly patrilineal, patrilocal and exclusively Islamic community. The women of Mitini are clearly seeking out genealogical kin. Note however that because of marriage rules that allow—even encourage—unions with both maternal and paternal cross cousins and with paternal parallel cousins, women’s affinal kin are often also genealogical kin. In other words, women’s in-laws are also their natal kin. In contrast to women, the men of Mitini make their village-level ties with more distantly related kin and with non-kin, favouring as co-workers those with whom they are friends and those with whom they have reciprocal relationships, irrespective of hamlet co-residence or genealogical relatedness. These gender differences may in part be driven by the greater specialization in economic activities among men (clove production, fishing, religious administration) than among women, who spend much time in pursuits in which all women engage (childcare, water collection, food preparation), or who can conduct distinct activities (weaving, hair plaiting, food production) in each others’ company.

Remarkably, women do not appear to be constrained in their relational ties to those of their husbands or male household members, suggesting greater gender autonomy than religious and cultural narratives might suggest. This is consistent with previous ethnographic work in Pemba [82], which emphasizes the term *tumbo*, an ego-focused and highly labile category of relatives. A woman’s understanding of *tumbo* is often more genealogically restricted than that of a man, and is often distinct from that of her husband. In a context of relatively high marital instability, where divorced women must depend on their natal lineage for support, such strategic reckoning allows for tensions between matrikin, patrikin and in-laws to be resolved, as [82] proposes. Taken together, the importance of gender for patterning relational wealth in the present analysis contributes to an emerging literature on gender differences (and similarities) in cooperation in rural communities [101]. More specifically, our results reflect previous findings that women are more active in maintaining larger networks of relational wealth *within their community* and are more likely to associate with kin [102], and that men are more likely to form relationships based on the material wealth of their partners [103].

### (d) Concluding remarks

In the absence of any clear wealth homophily, there is as yet little evidence of emerging cleavages in relational and material wealth that can exacerbate wealth inequality, as so widely reported in the literature. Despite the opportunities for highly unequal cash windfalls that are contingent on sharp differences in tree ownership (cloves and other), there appear to be few pathways whereby wealth inequality will become entrenched or socially divisive in Mitini. This is not necessarily true of Pemba more generally, given that we selected a village for study that had no ties of remittance with relatives in Oman or other foreign lands. However, in Mitini, we suggest—albeit through inference and ethnographic knowledge—that it is the unpredictability of cash income that drives everyone to buffer their fortunes with relational wealth, and benefit from the strong prescriptions for interdependence among neighbours, as prescribed by Islam. Perhaps the only emerging cracks in the fabric are that men are clearly carefully selecting co-workers based on reciprocity rather than genealogical relationship or distance, and that a small clique may be emerging among households with *Nguvu Zetu* members that share a somewhat positive orientation towards development. These patterns may take on a new significance in the future. Examining this patterning will allow us to understand how persistent inequality emerges between regions, groups and populations [104], and might indicate where and how development interventions can most effectively reduce inequality [20].

More generally, bridging individual-level and macro-level structural factors, core meso-level forces—such as social networks—provide a platform for gaining a deeper understanding of mechanisms leading to material wealth inequality. We suggest that the type of study presented here, that attempts to disaggregate theoretically relevant features of relational wealth and how they are related to material wealth, will allow exploration of how individual-level processes aggregate up to form the norms and conventions that, in turn, feedback into individual differences in access to material resources.

## Data Availability

All data and code for processing and analysing data are publicly available from the Dryad Digital Repository: https://doi.org/10.5061/dryad.rr4xgxddf [105]. The data are provided in the electronic supplementary material [106].
